# A rare *de novo* interstitial duplication of 15q15.3q21.2 in a boy with severe short stature, hypogonadism, global developmental delay and intellectual disability

**DOI:** 10.1186/s13039-016-0214-3

**Published:** 2016-01-11

**Authors:** Haiming Yuan, Zhe Meng, Lina Zhang, Xiangyang Luo, Liping Liu, Mengfan Chen, Xinwei Li, Weiwei Zhao, Liyang Liang

**Affiliations:** Sun Yat-Sen Memorial Hospital, Sun Yat-Sen University, Guangzhou, 510120 Guangdong China; Guangzhou kingmed center for clinical laboratory Co.,Ltd., Guangzhou, 510330 Guangdong China; KingMed School of Laboratory Medicine Guangzhou Medical University, Guangzhou, 510330 Guangdong China; Department of Obstetrics and Gynecology, Wuhan women and children medical healthcare center, Wuhan, 430016 China

**Keywords:** Short stature, Hypogonadism, Global developmental delay, Intellectual disability, Interstitial duplication15q15.3q21.2

## Abstract

**Background:**

Interstitial duplications distal to 15q13 are very rare.

**Case Presentation:**

Here, we reported a 14-year-old boy with severe short stature, delayed bone age, hypogonadism, global developmental delay and intellectual disability. His had distinctive facial features including macrocephaly, broad forehead, deep-set and widely spaced eyes, broad nose bridge, shallow philtrum and thick lips. A *de novo* 6.4 Mb interstitial duplication of 15q15.3q21.2 was detected by chromosomal microarray analysis. We compared our patient’s clinical phenotypes with those of several individuals with overlapping duplications and several candidate genes responsible for the phenotypes were identified as well.

**Conclusion:**

The results suggest a novel contiguous gene duplication syndrome characterized with shared features including short stature, hypogonadism, global developmental delay and other congenital anomalies.

**Electronic supplementary material:**

The online version of this article (doi:10.1186/s13039-016-0214-3) contains supplementary material, which is available to authorized users.

## Background

Microdeletions and microduplications are recurrent for the proximal region of 15q. Five common breakpoints (BP1–BP5) exist in the 15q11.2-q13 region. Deletions between BP1–BP3 are responsible for the Prader-Willi and Angelman syndromes, depending on the parent-of-origin, whereas the reciprocal duplications of the region, particular the gains of maternal copy of 15q11.2-q13, are known as 15q duplication syndrome (Dup15q) characterized by global developmental delay, autism spectrum disorder and epilepsy [1–3]. 15q13.3 microdeletions and microduplications between BP4–BP5 are enriched in neurodevelopmental and neuropsychiatric disorders [4, 5]. On the other end of the 15q, the terminal trisomy or tetrasomy of 15q, including *IGF1R* gene, is named as 15q26 overgrowth syndrome consisting of overgrowth, learning difficulties, distinctive facial features and multiple congenital anomalies [6]. The monosomy of 15q26 shows the opposite growth features, such as pre- and postnatal growth retardation, as well as some different dysmorphic features [7]. A few proximal microdeletions at 15q25.2 have been reported to be associated with congenital diaphragmatic hernia, cognitive deficits and Diamond-Blackfan anemia and some more distal deletions to neurodevelopmental and neuropsychiatric disorders [8–11]. Whereas duplications distal to 15q13 are very rare. Elcioglu (1997) described a patient with hypogonadism, skeletal anomalies, Marfan-like features, developmental delay and intellectual disability, who carried a *de novo* interstitial inverted duplication involving bands of 15q13.3-q21.3 [12]. Herr (1983) reported a patient with a *de novo* interstitial duplication of 15q14-q21.1, who presented with hypogonadism, skeletal problems, short stature, delayed bone age, global developmental delay and intellectual disability [13]. Both of the duplications were uncovered by high-resolution G-banded cytogenetics analysis, so the precise locations and sizes of the duplications were not determined [12, 13]. Here, we reported a *de novo* 6.4 Mb interstitial duplication of 15q15.3q21.1 in a 14-year-old boy with hypogonadism, short stature, global developmental delay and multiple congenital anomalies.

## Case presentation

The proband is the fourth child of healthy unrelated parents with negative family history. His siblings are all healthy. Intrauterine growth retardation was noticed by ultrasound examination at 7 months of pregnancy. He was born by vaginal delivery at 38 weeks of gestation. Birth weight was 2.9 kg (<-1SD), length 47 cm (<-2SD) and head circumference 34 cm. Apgar scores were all 9. No feeding difficulty was noted at all times.

**Fig. 1:**
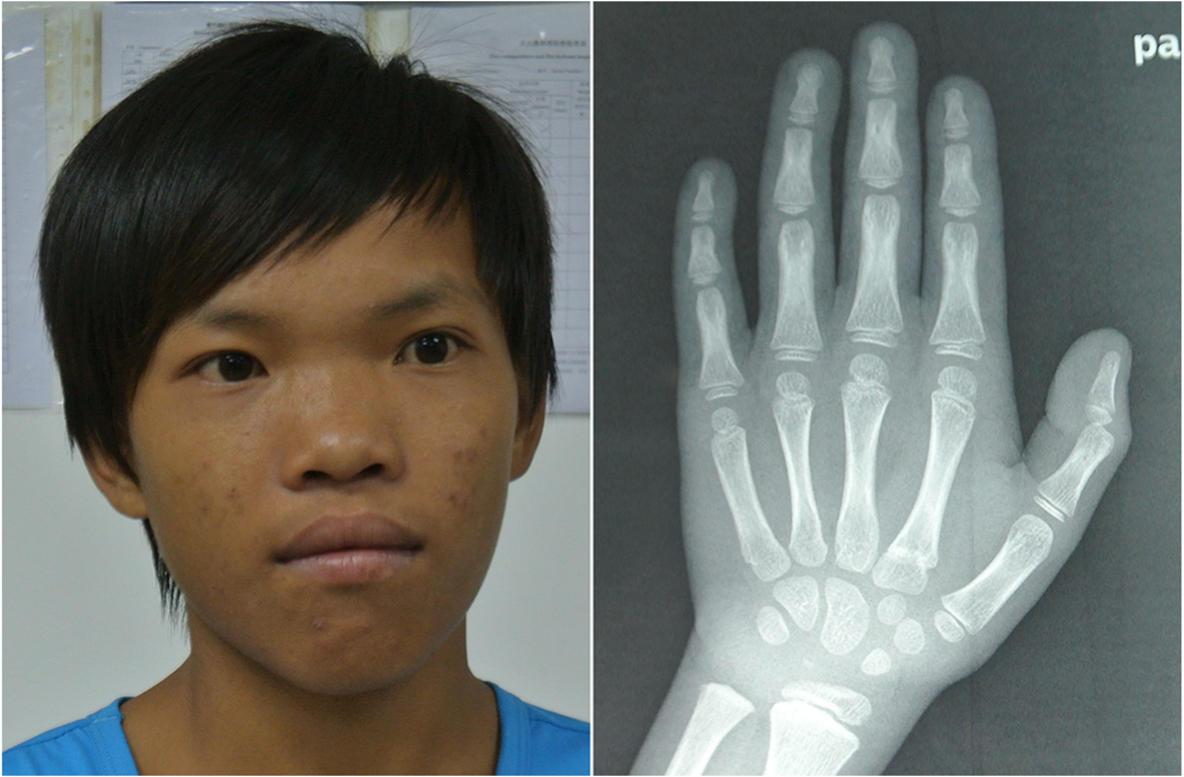
The probandat 14 years of age. Note relative macrocephaly, coarse face, broad forehead, deep-set and widely spaced eyes, strabismus, broad nose bridge, shallow philtrum, thick lips. Hand X-ray showed delayed bone age.

The development milestones were delayed: he raised his head at 4 months of age, sat at 9 months and independently walked at 1 year 6 months. Language development was significantly delayed. The patient was examined at the age of 14 years. His height was 140 cm (<-2SD), weight 33.5 kg (<-2SD) and head circumference 54 cm, which indicated persistent failure to thrive. He had moderate intellectual disability and showed poor performance in the elementary school. His distinctive facial features were characterized by macrocephaly, coarse face, broad forehead, deep-set and widely spaced eyes, strabismus, broad nose bridge, shallow and short philtrum and thick lips. He had severe short stature and hands X-ray showed delayed bone age (Fig. 1). Yet his growth hormone level (6.19 ng/ml) and level after provocation tests (11.6 ng/ml) were all normal. Pituitary magnetic resonance imaging and thyroid hormone level were also normal. Micropenis, small testes and low testosterone were detected. No additional abnormalities was noticed.

## Methods

### Chromosomal microarray analysis

Chromosomal microarray analysis was performed for the proband and both parents by Affymetrix Cytoscan HD Array (Affymetrix, USA). Genomic DNA was extracted from peripheral blood using a commercial kit (Qiagen). The labeling and hybridization procedures were performed following manufacturer’s instructions. The raw data of chromosomal microarray was analyzed by Affymetrix Chromosome Analysis Suite Software.

### Confirmation of 15q15.3q21.2 duplication

The duplication was further confirmed using quantitative real-time PCR analysis. Primer sequences and descriptions were included in Additional file 1: Table S1.

## Results

**Fig. 2:**
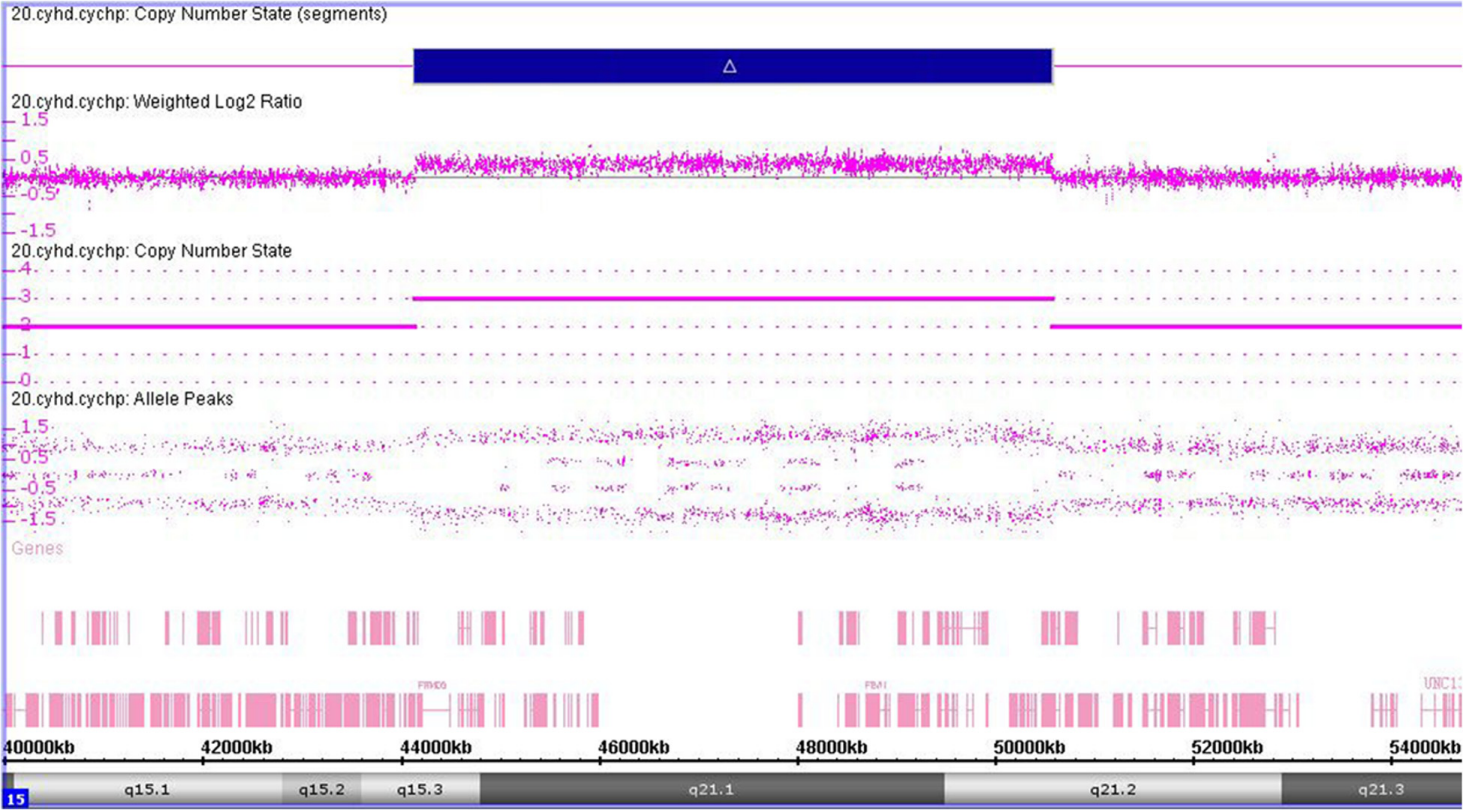
Affymetrix cytoscan HD array analysis including weighted log2 ratio (upper), copy number state (middle) and allele peaks (lower) are shown for chromosome 15. The result shows duplication at 15q15.3q21.2. The genomic coordinates (hg19): chr15:44,143,547–50,572,601. The copy number gain region is denoted by a blue bar

A 6.4Mb duplication at 15q15.3q21.2 (chr15: 44,143,547–50,572,601) was detected by CMA (Fig. 2). Parental tests were normal. Thus, the proband carried a *de novo* copy number variant. The duplication was further confirmed by quantitative real-time PCR analysis (data not shown).

## Discussion

**Fig. 3:**
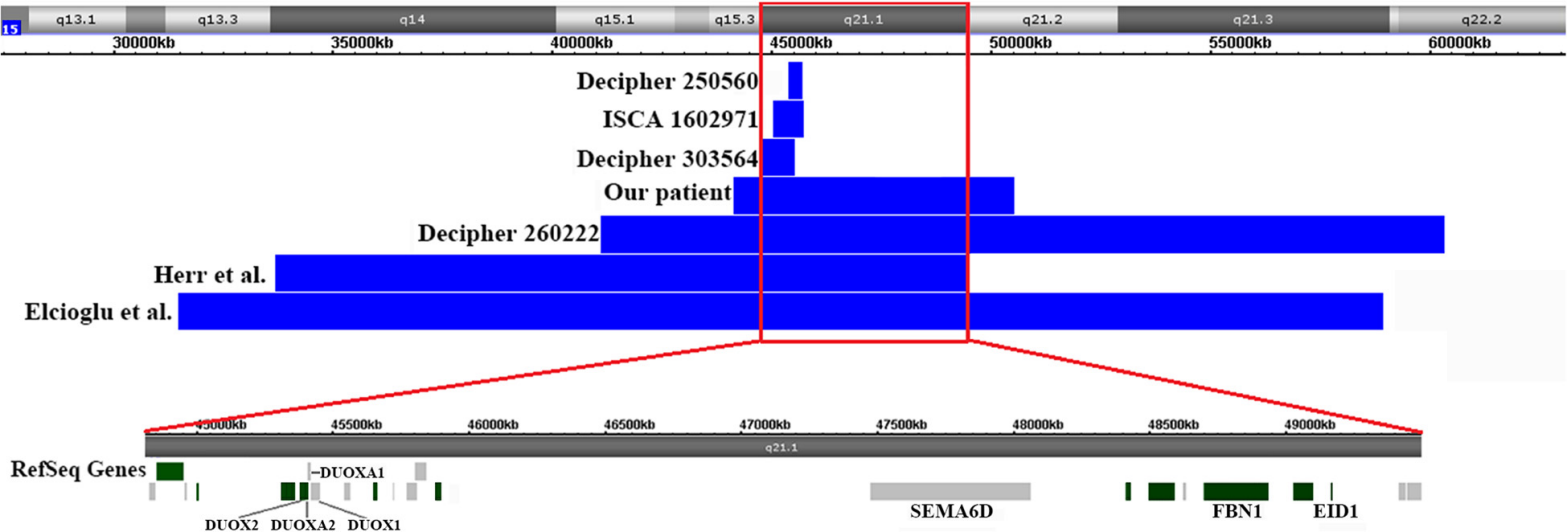
Top panel shows a genome view of all duplication cases (blue colored custom tracks) relative to the genomic coordinates at 15q13.3q22 region, extracted from Human Genome Build 37 (hg19). Red block diagram represents critical region of 15q21.1. Bottom panel shows the zoomed-in 15q21.1 region encompassing candidate genes. Note: Two patients reported by Herr and Elcioglu were detected by high resolution G-banding analysis, therefore the genomic coordinates of the relevant bars were approximate locations.

Interstitial microdeletions and duplications involving 15q except for the 15q11-q13 region were very rare. Here, we reported a 14-year-old boy with severe short stature, delayed bone age, hypogonadism, global developmental delay, intellectual disability and distinctive facial features, who carried a *de novo* 6.4 Mb duplicationon 15q15.3q21.2. No other clinical significant CNVs were detected in this patient. No duplication at this interval was reported in DGV. It was reasonable to suggest that this *de novo* duplication was likely pathogenic and mostly responsible for the patient’s clinical condition. Next we searched databases for duplications overlapping with this region. We identified 3 cases from DECIPHER, 1 case from ISCA, 2 cases from literatures. The locations and sizes of these duplications are depicted in Fig. 3 and their clinical information detailed in Table 1.

**Table 1:** Genomic and clinical information of patients with duplication of 15q13.3q22. The genomic coordinates are based on GRCH37/hg19

Reference	Our patient	Decipher 260222	Herr et al., 1983	Elcioglu et al., 1997	Decipher 250560	ISCA nssv1602971	Decipher 303564
Sex	male	male	male	male	male	unknown	female
Genomic location	chr15:44143547-50572601	chr15:41140284-60397505	chr15q14q21.1	chr15q13.3q21.3 inverted duplication	chr15:45404526-45733361	chr15:45058112-45769052	chr15:44792878-45568844
Size	6.4Mb	19.3Mb	about 15Mb	about 25Mb	330Kb	711Kb	780Kb
Methods	microarray	microarray	high resolution G-banding analysis	high resolution G-banding analysis	microarray	microarray	microarray
Origin	*De novo*	unknown	*De novo*	*De novo*	inherited from parent with similar phenotype	unknown	unknown
Phenotype	hypogonadism,	hypogonadism,	hypogonadism,	hypogonadism,	ID, language delay,	DD, MCA	Cognitive
	short stature,	short stature,	short stature,	skeletal problems,	macrocephaly,		impairment
	delayed bone age,	delayed bone age,	skeletal problems,	Marfan-like features,	muscular hypotonia		
	language delay	delayed cranial suture closure,	language delay,	language delay,			
	ID, DD,	language delay,					
	distinctive facial features, macrocephaly,	ID, DD,	ID, DD,	ID, DD,			
		seizures	distinctive facial features, seizure	macrocephaly			
Endocrine examine	thyroid and growth hormone are normal	NA	thyroid and growth hormone are normal	NA	NA	NA	NA

*Abbreviation*: *ID* intellectual disability; *DD* developmental delay; *MCA* Multiple congenital anomalies; *NA* not available

Elcioglu (1997) [12] and Herr (1983) [13] each reported a case with a *de novo* interstitial duplication on 15q13.3q21.3 and 15q14q21.1 respectively. The Decipher case 260222 largely overlapped with the duplication detected in our patient. Our patient had the smallest duplication among these four patients with large duplication centered on 15q21.1. We noticed that three patients (our patient, Decipher case 260222 and the patient reported by Herr) had strikingly similar clinical features including hypogonadism, short stature, delayed bone age, global developmental delay and intellectual disability. Interestingly, the patient reported by Elcioglu shared most of similar phenotypes, but presented with Marfan-like features such as tall stature. All duplications were *de novo* except for Decipher case 260222 without parental tests and not reported in DGV database, as well as these patients had similar clinical conditions especially hypogonadism and skeletal problems, which further suggested a likely pathogenic nature of these duplications.

To further support this notion, we identified several candidate genes responsible for complex clinical phenotypes. The *FBN1* (134797) gene is involved in these duplications. As we know that different mutations of *FBN1* gene cause different dominant genetic diseases characterized with opposite stature. For example, acromicric dysplasia caused by *FBN1* mutations is characterized by severe short stature, short limbs, delayed bone age, stiff joints and facial dysmorphism [14]. Weill-Marchesani syndrome caused by *FBN1* mutations consists of short stature, eye abnormalities, unusually brachydactyly, joint stiffness and heart disease [15]. Marfan syndrome caused by *FBN1* mutations on the other hand is charaterized by tall and slender stature with arachnodactyly, as well as ocular and cardiovascular defects [16]. Thus, skeletal anomalies could be explained by *FBN1* gene duplication at this interval.

Other than the *FBN1* gene that was known to cause skeletal problems, no other known disease gene at this interval could explain all clinical phenotypes of these patients. Smaller overlapping duplications shared by Decipher cases 250560 and 303564 and an ISCA case 1602971 involved the NOX family genes such as *DUOX2*, *DUOXA2*, *DUOXA1* and *DUOX1*. The loss of function mutations in these genes could cause permanent congenital hypothyroidism [17–20]. However, thyroid examinations of our patient and the one reported by Herr were both normal and other patients were lack of this information, it may be inferred that the clinical consequence of genes duplication was not known. And there was no additional evidence to support the pathogenicity of these small duplications.

Among other genes at the interval, we identified the *EID1* gene as an interesting candidate gene. *EID1* was shown to be expressed ubiquitously in human tissues with high expression in neuron, cardiac and skeletal muscle [21, 22]. Liu et al. generated an *EID1* transgenic mouse model that exhibited neuron-specific overexpression of human *EID1* gene in the brain, and overexpression of *EID1* reduced hippocampal long-term potentiation and impaired spatial learning and memory function [23]. *EID1* tightly interacted with CBP/p300 in the nuclei and had been identified as an inhibitory protein of CBP/P300 [23]. CBP/p300, a family of CREB-binding proteins, was associated with Rubinstein–Taybi syndrome characterized by short stature, intellectual disability, developmental delay and distinctive facial features [24]. Overexpression of *EID1* in human brain could negatively regulate CBP/p300 activity and lead to the impairment of neuronal function. In addition, EID-1 was known to be co-expressed with the Steroidogenic factor-1 (SF-1) which played a critical role in adrenal and reproductive development and function [25, 26]. Park et al. provided convincing evidence demonstrating that *EID-1* strongly inhibited the transcriptional activity of SF-1 [27]. It was tempting to speculate that duplication of *EID-1* may also be responsible for the hypogonadism phenotype.

Finally, we identified the *SEMA6D* gene as another candidate gene. *SEMA6D* gene, one member of the class VI subgroup of the semaphorin family, was expressed abundantly in kidney, brain, and placenta and moderately in the heart and skeletal muscles. The expression of *SEMA6D* predominantly in the adult tissues made them potentially important molecules in nervous system maintenance and repair. This profile was consistent with a more general role of the proteins in neurogenesis and organogenesis as well as in regenerative and degenerative processes [28]. Therefore, we hypothesized that overexpression of *SEMA6D* may play a role in neurodevelopmental anomalies of our patient.

In conclusion, we reported a *de novo* duplication of 15q15.3q21.2 in a patient with skeletal problems, hypogonadism, global developmental delay, intellectual disability and facial dysmorphism. Several candidate genes responsible for the complex clinical features were identified and critical region centered on 15q21.1 was defined as well, thus we proposed a novel contiguous gene duplication syndrome at this genomic interval. Further studies on duplications involving this region will be necessary.

## Consent

Written informed consent was obtained from the patient for publication of this case report and any accompanying images. A copy of the written consent is available for review by the Editor-in-Chief of this journal.

## Additional file

Additional file 1: Table S1. qPCR confirmation of CMA results. (DOCX 22 kb)
